# Pattern and determinants of endometrial thickness among asymptomatic postmenopausal women in an African population

**DOI:** 10.4314/ahs.v24i4.17

**Published:** 2024-12

**Authors:** Tola Y Bakare, Adegboyega A Fawole, Kikelomo T Adesina, Hadijat O Raji, Bola B Olafimihan, Abiodun S Adeniran

**Affiliations:** 1 Department of Obstetrics & Gynaecology, Bowen University, Iwo / Bowen University Teaching Hospital, PMB 284, Iwo, Nigeria; 2 Obstetrics & Gynaecology Department, University of Ilorin/ University of Ilorin Teaching Hospital, PMB 1515, Ilorin, Nigeria; 3 Department of Radiology, University of Ilorin Teaching Hospital, PMB 1459, Ilorin, Nigeria

**Keywords:** Endometrial thickness, postmenopausal women, biosocial characteristics, determinants, asymptomatic women

## Abstract

**Background:**

The occurrence of endometrial cancer may be on the increase among African population due to lifestyle changes. Measurement of endometrial thickness (ET) in postmenopausal women may enhance timely diagnosis of endometrial pathology to improve prognosis and quality of life.

**Objectives:**

To determine the relationship between anthropometric measurements hypertension and diabetes mellitus on ET.

**Methods:**

A cross-sectional study conducted at the outpatient clinics of a tertiary facility among asymptomatic postmenopausal women (no malignancy, abnormal vaginal bleeding or hormone replacement therapy). Participants recruited by purposive sampling were sub-categorized into those with chronic hypertension, diabetes mellitus or no chronic medical disorder. All participants had anthropometric measurements, fasting blood glucose and transvaginal ultrasonography. The main outcome measure was the ET while p-value <0.05 was significant.

**Results:**

The mean ET was 2.17±2.57 and prevalence of ET (>5mm) was 1.1%. Mean ET was significantly higher among women <5 years post-menopause (2.53±1.61 vs. 2.06±2.79; P0.048); chronic hypertension (2.82±4.07mm vs. 1.42±1.16mm; P0.026) or diabetes mellitus (2.27±1.08mm vs. 1.42±1.16; P0.005). Parity was inversely related to ET (P0.005); body mass index (P0.191), duration of hypertension (P0.213) or diabetes mellitus (P0.085) were not statistically significant.

**Conclusion:**

Parity, number of years post-menopause, hypertension and diabetes mellitus were important determinants of ET.

## Introduction

Endometrial cancer is the commonest gynaecological cancer in high income countries and the third commonest in sub-Sahara Africa.^1^ An endometrial thickness (ET) of <5mm is regarded as normal for postmenopausal women while an association has been established between ET and endometrial diseases.^1^ However, 10.5mm ET was recommended as the cut-off for postmenopausal women on hormone replacement therapy before further evaluation using endometrial biopsy and histology.^2^ However, literature search reveal paucity of data on the association between chronic hypertension, diabetes mellitus and body mass index (BMI) on ET in sub-Sahara Africa.

The endometrium is a dynamic tissue in the life of a female, it undergoes cyclical changes regulated by oestrogen and progesterone. The ET varies according to the phases of the menstrual cycle via oestrogen and progesterone receptors which respond to the hormones. Excessive oestrogen may lead to endometrial hyperplasia and its paucity causes endometrial atrophy. Although high resolution transvaginal ultrasonography (TVS) is important in assessing the endometrium, a thickened endometrium is always a clinical conundrum. Again, there remains variation on the cut-off of ET which requires intervention based on study reports.^3^

Although the ovaries are the major source of oestrogen, peripheral production in adipose tissue also contributes and this is influenced by medical disorders like metabolic X syndrome. Obesity is associated with insulin resistance with resultant high plasma level of insulin, which increases free oestrogen levels by decreasing the concentration of sex hormone binding globulin that normally acts as carrier for oestrogen and other sex hormones in the blood.^4^ Also, Insulin growth factor (IGF-1) and its binding protein (IGF binding protein-1) promote endometrial cell growth; thus, the high levels of IGF found in diabetic women and women with higher BMI may theoretically predispose to endometrial hyperplasia.^5^ TVS with or without colour flow imaging with measurement of maximal endometrial thickness on a midline sagittal image of the uterus has been recommended as a screening tool for endometrial cancer.^1^

Asymptomatic endometrial thickening defined as an ET >5mm on ultrasonography in a postmenopausal woman without vaginal bleeding poses a clinical dilemma relative to the need or otherwise for further evaluations.^6^ Although postmenopausal endometrial thickness >5mm may suggest malignancy, the thickness depends on age, parity, menopausal years, BMI, chronic medical illness (e.g. diabetes mellitus or hypertension), drugs (e.g. tamoxifen) or hormone replacement therapy (HRT).^7^ These factors exert their influence on the endometrium and the resultant changes in the endometrial lining may be measured on ultrasonography. This study was aimed at describing the association of anthropometric measurements and chronic medical disorders (hypertension and diabetes mellitus) with postmenopausal endometrial thickness.

## Materials and methods

This was a prospective cross-sectional study conducted at University of Ilorin Teaching Hospital (UITH), Ilorin, Nigeria; participants were asymptomatic postmenopausal women attending the specialist outpatient clinics (gynaecology, internal medicine and family medicine). Participants were divided into 3 groups of those with hypertension only, those with diabetes mellitus only and those without any known chronic medical disorders. The inclusion criteria were postmenopausal women (menopause was defined from one year from last menstrual period), women diagnosed with diabetes mellitus or hypertension for a minimum of one year and asymptomatic women with no chronic medical disorder who consented to participate in the study while women who had hysterectomy or oophorectomy (surgical menopause), postmenopausal vaginal bleeding, women diagnosed with female genital malignancy (endometrial, cervical or vaginal cancer) were excluded from the study. Eligible women were counseled and a written informed consent was obtained. Thereafter, relevant history was obtained including parity, age at menopause, duration of menopause (years since menopause) and associated medical illnesses (hypertension or diabetes mellitus).

### Measurements undertaken in the study

Anthropometric measurement was obtained by measuring the weight using a ZT-120 weighing scale to the nearest 0.1kg with participants in minimum clothing while the height was measured to the nearest 0.1 metre using a portable stadiometer with the participant barefooted. BMI was computed as (weight [kg] / (height [m]2). A BMI of <18.5kgm-2 was regarded as underweight, 18.5-24.9kgm-2 as normal, 25.0-29.9kgm-2 as overweight and ≥30kgm-2 as obese.

Systolic and diastolic blood pressures were measured using a mercury Sphygmomanometer with participants in a sitting position. Hypertension was defined as systolic value ≥140mmHg or diastolic ≥90mmHg on two occasions at least six hours apart in a previously normotensive individual.

Blood sample for fasting blood glucose was obtained from all participants after an overnight fast of at least 8 hours using a standardized glucometer to exclude pre-existing undiagnosed diabetes in the participants.

All participants had transvaginal ultrasound scan (TVS) to assess endometrial thickness using Aloka SSD-1000 ultrasound machine designed by ALOKA GmbH, Meerbusch, Germany using a frequency of 7.5MHZ. The participants first emptied the urinary bladder, then in dorsal position and draped to expose only the perineum in the presence of a female chaperon. The transvaginal probe was lubricated with the coupling gel and covered with a male condom; the lubricated probe was inserted gently into the vagina and with gentle manipulation. The endometrial thickness was measured in the longitudinal (sagittal) plane between the two basal layers of the anterior and posterior uterine wall at the thickest point. The probe was removed from the vagina after measurement; the condom protection cap was reoved and cleaned in between each use to prevent cross infections. The only woman with an abnormal thickened endometrium was counselled; she had endometrial biopsy using manual vacuum aspiration for histology. Two trained personnel were present and took each measurement using the study protocol, the harmonized measurement was assigned to limit inter and intra-observer errors.

### Sample size determination

The sample size for this study was calculated using the formula below:


$$\begin{array}{ccc} n & = & \left(\left(z\alpha +z\beta \right)\sigma \right)2\\ & & \;\;\;\left(µ1-µ0\right)2 \end{array}$$

Where: zα = 1.96 (critical value dividing central 95% of z distribution from 5% in the tails)

zβ = 1.28 (critical value that separates the lower 10% of distribution from upper 90%)

σ = SD

µ1 - µ0 = difference of two means.

Using a previous prospective study on asymptomatic postmenopausal women using TVS for ET with mean 3.8±2.3mm.^8^ This reference study was used to calculate the sample size due to unavailable local values as well as the similarity in the objectives and methodology of both studies.

The sample size was estimated to show the significant difference in means at 1 mm

n1= (1.96 + 1.28)2 (2.3)2 = 56

(1)2

With a provision of 10% for attrition (6 participants), the minimum sample size for the study was (56+6) = 62 participants. However a total number of 89 participants were recruited to enhance the power of the study.

### Categorization of participants and sampling method

Participants in the study were categorized into three groups of those with chronic hypertension, those with diabetes mellitus and those without any chronic medical disorder with near equal numbers of participants to allow for proper representation. The sampling technique employed was purposive sampling in which all consecutive consenting participants were recruited into the study until the sample size was completed.

### Data analysis

The data was analyzed using the Statistical Package for Social Sciences software (SPSS) version 23.0; endometrial thickness was found to be skewed (skewness z score 24.0314), thus it was analyzed with Mann-Whitney U test and Kruskal Wallis test. Probability (p) values less than 0.05 was accepted as statistically significant.

### Ethical approval/consent

Ethical approval was obtained from the ethical review committee of the tertiary hospital (Approval number ERC PAN/2016/02/1492) before commencement of the study. Also, a written informed consent was obtained from each participant at recruitment.

## Results

A total of 120 women were screened for the study out of which 89 eligible participants were recruited and categorized into 30(33.7%) with chronic hypertension, 29(32.6%) with diabetes mellitus and 30(33.7%) without any known chronic medical disorder. Table 1 show that the participants were aged 51-82 years with mean ages for participants with hypertension (65.67years±7.68), diabetes mellitus (67.79±8.79 years) and no medical disorders (61.30 years±7.34). The mean age at menopause was lowest for participants without medical disorders (48.33±2.29 years) and highest for participants with diabetes mellitus (50.14±3.99 years) while the mean years since menopause was 12.97±6.69 years for women without chronic disorders, 16.40±6.75 years for women with hypertension and 17.66±7.76 years for women with diabetes mellitus.

**Table 1:** Biosocial characteristics of the participants

Variables	Hypertension n=30(%)	Diabetes n =29(%)	No medical disorder n =30(%)
**Age group (years)**			
51 – 60	8 (24.2)	7 (21.2)	18 (54.6)
61 – 70	14 (41.2)	12 (35.3)	8 (23.5)
> 70	8 (36.4)	10 (45.4)	4 (18.2)
Mean ± SD	65.67 ± 7.68	67.79 ± 8.79	61.30 ± 7.34
Range	54 – 80	54 – 82	51 – 75
**Marital status**			
Single	1 (50.00	1 (50.0)	0 (0.0)
Married	24 (33.3)	22 (30.6)	26 (36.1)
Widowed	5 (33.3)	6 (40.0)	4 (26.7)
**Occupation**			
Unemployed	2 (50.0)	0 (0.0)	2 (50.0)
Artisan	3 (37.5)	2 (25.0)	3 (37.5)
Trader	16 (32.7)	16 (32.7)	17 (34.6)
Civil Servant	10 (35.7)	10 (35.7)	8 (28.6)
**Education**			
None	1 (11.1)	6 (66.7)	2 (22.2)
Primary	9 (42.8)	6 (28.6)	6 (28.6)
Secondary	12 (41.4)	7 (24.1)	10 (34.5)
Tertiary	8 (26.7)	10 (33.3)	12 (40.0)
**Parity**			
0	1 (33.3)	2 (66.7)	0 (0.0)
1	1 (50.0)	0 (0.0)	1 (50.0)
2 – 4	15 (40.6)	9 (24.3)	13 (35.1)
≥5	13 (27.7)	18 (38.3)	16 (34.0)
**Age at menopause (years)**			
Mean ±SD	49.27±3.48	50.14±3.99	48.33±2.29
Range	47-58	45-58	45-58
**Years passed since menopause**			
≤5	6(22.2)	6(22.2)	15(55.6)
6-10	16(42.1)	13(34.2)	9(23.7)
>10	8(33.3)	10(41.7)	6(25.0)
Mean ± SD	16.40±6.75	17.66±7.76	12.97±6.69
Range (years)	5-30	7-31	1-25
**Previous menstrual irregularity**			
Yes	6(28.6)	11(52.4)	4(19.0)
No	24(35.3)	18(26.5)	26(38.2)
**Chronic medical disorder (mean duration) in years**	15.37±9.94	12.21±6.38	
Range	1-34	3-30	

Table 2 shows that the study's overall mean ET was 2.17±2.57mm (range 0.10 to 23.00), mean weight was 72.24±14.60kg (range 46 to 106) and mean height of 1.58±0.06m. Also, 43(48.4%) were obese (BMI ≥30kg/m2) while the BMI ranged from 19.15 to 41.60kg/m2. ET <5mm was observed in one participant representing 1.1%.

**Table 2:** Endometrial thickness and anthropometric parameters of the study population

Variables	Frequency	Percent
**Endometrial thickness (mm)**		
< 5	88	98.9
≥ 5	1	1.1
Mean ± SD	2.17 ± 2.57	
Range	0.10 – 23.00	
**BMI (kg/m^2^)**		
18.5 – 24.9	37	41.6
25.0 – 29.9	9	10.1
30.0 – 34.9	28	31.5
35.0 – 39.9	11	12.3
≥ 40	4	4.5
Mean ± SD	29.01 ± 5.98	
Range	19.15 – 41.60	
**Weight (kg)**		
Mean ± SD	72.24 ± 14.60	
Range	46.00 – 106.00	
**Height (m)**		
Mean ± SD	1.58 ± 0.06	
Range	1.48-1.80	

Table 3 shows that the ET was significantly higher in women who were ≤5 years compared to women >5years post menopause (2.53±1.61 vs. 2.06±2.79; P 0.048) and inversely related to the parity (P 0.005). The ET was significantly higher among women with hypertension (2.82±4.07 vs. 1.42±1.16; P 0.026) and diabetes (2.27±1.08 vs. 1.42±1.16; P 0.005) compared to those without chronic medical disorders. The duration of the hypertension (P 0.213) or diabetes (P 0.085) and the BMI (P0.191) were not significantly associated with ET.

**Table 3:** Relationship between endometrial thickness and individual parameters of participants

Variables	Endometrial thickness (mm) Mean ± SD	Test	*p* value
**Post-menopause (years)**			
≤ 5	2.53 ± 1.61	488.500^U^	0.048*
> 5	2.06 ± 2.79		
**Parity**			
0	2.70 ± 1.56	12.890^K^	0.005*
1	2.55 ± 0.21		
2 – 4	2.45 ± 1.26		
> 4	1.89 ± 3.34		
**Previous menstrual abnormalities**			
Yes	2.36±1.21	546.500^U^	0.105
No	2.10±2.87		
**Hypertension versus Nil medical disorders**	2.82 ± 4.07 vs. 1.42±1.16	299.500^U^	0.026
**Diabetes versus Nil medical disorders**	2.27 ± 1.08 vs. 1.42±1.16	256.00^U^	0.005
**Body Mass Index (kg/m^2^)**			
18.5-24.9	1.58±1.21	4.746^K^	0.191
25.0-29.9	1.61±1.02		
30.0-34.9	2.33±1.64		
35.0-39.9	2.38±1.28		
≥40.0	2.70±3.68		
**Duration of hypertension (years)**			
<10	3.93±5.52	76.500^U^	0.213
≥10	1.81±1.07		
**Duration of diabetes mellitus (years)**			
<10	2.51±1.24	70.000^U^	0.085
≥10	1.85±0.89		

U **Mann Whitney U test**

K **Kruskal Wallis test**

Table 4 shows a statistically significant correlation between ET and the weight (P0.021) while BMI (P0.111) and height (P0.793) were not statistically significant.

**Table 4:** Correlation of relationship between endometrial thickness and anthropometric measurements

Variable	Endometrial thickness R	*p* value
BMI	0.174	0.111
Weight	0.251	0.021*
Height	0.029	0.793

**r: Spearman correlation coefficient**

* ***p* value <0.05 (i.e. statistically significant)**

## Discussion

A total of 89 asymptomatic postmenopausal women including those with or without chronic medical disorders (hypertension or diabetes mellitus) participated in the study. The prevalence of asymptomatic ET was 1.1% and is similar to 2.4% from a similar study in Nigeria.^9^ This contrasts with 50.8% from China by Xue et al^10^ using cut-off of 5mm while Ozelci et al^11^ in Turkey using series of cut-off values reported prevalence of 57.1% (6-10mm), 26.3% (11-15mm), 10.5% (16-20mm) and 6.1% (>20mm). This variation may be due to differences in the cut-off value, lifestyle and geographical location of the study population.

Again, interest has been growing on the implication of increased ET on further action and the association with endometrial pathology. In this study, one participant with ET >5mm had endometrial biopsy with histology showing complex adenomatous hyperplasia for which she had total abdominal hysterectomy plus bilateral salpingo-oophorectomy. In a similar study which assessed association between ET and endometrial pathology, the authors reported atypical endometrial hyperplasia (AEH) of 0% (6-15mm), 7.1% (16-20mm) and 12.5% (>20 mm) respectively.^11^ However, a diagnosis of endometrial carcinoma was made in 3.5% of asymptomatic women with ET 6-10mm. The authors recommended further evaluation for women with ET cut-off of 10.5mm or lower ET values in the presence of other risk factors.^11^ In a similar study, 7.4% of women with ET had endometrial hyperplasia^10^ while another study reported benign histology report in 72.2% of participants, pre-malignant lesions in 18.1% and malignant lesions in 9.7%.^12^ Another study reported an increasing probability of endometrial malignancy from 1.012-fold (ET 5-8mm), 1.769 (ET 8-11mm) and 4.737-fold (ET >11mm).^13^ In addition, Zhang et al reported that ET was significantly correlated with atypical hyperplasia and endometrial cancer with an odd of 1.252 using optimal ET cut-off of 8mm.^14^. These suggests that in the face of the variation in the cut-off values of ET, all women with asymptomatic ET shoulde considered for further evaluation to rule out endometrial pathology.^12^

The mean age at menopause in this study was comparable to reports of 49.36±5.0 years^15^ from Nigeria and about 50 years^16^,^17^ from other continents. Obesity is increasing globally, and it is a risk factor for endometrial hyperplasia and endometrial cancer. However, the obesity rate of 48.4% among postmenopausal women in this study is slightly higher than 42.2% in a similar study.^18^ Obesity among postmenopausal women has been linked to the menopausal transition characterized by reduced resting metabolic rate, lowered energy expenditure and increase in fat mass and central adipose tissue accumulation. Although there was no significant relationship between BMI and endometrial thickness in this study, there was a corresponding increase in endometrial thickness as BMI increases. However, reports from other studies are divergent ranging from no significant^19^ to statistically significant relationship between endometrial thickness and BMI.^20^ This difference may be attributed to the difference in methodology and BMI classification for example one study defined obesity as BMI > 25kg/m^2^.^20^

The mean ET decreased significantly with increasing years after menopause similar to a previous report.^21^ This is probably due to the progressive fall in hormone levels particularly oestrogen as the menopausal year increases. Endometrial thickness decreased as parity increased, with nulliparous participants having the highest ET. While low parity is related to longer period of exposure to unopposed oestrogen without disruption of the normal cycle, mechanical shedding of precursor cells of malignant potential at each delivery was reported to prevent thickened endometrium and related disorders in subsequent years.^22^ The anti-mitotic property of progesterone which is increased during pregnancy also limits endometrial growth^23^ thereby preventing unrestrained endometrial proliferation and the risk of endometrial malignancy.

Both hypertension and diabetes mellitus are part of the metabolic X syndrome associated with increased ET in postmenopausal women.^8^ The increased ET among women with hypertension in this study supports an earlier report 18 although others reported no statistically significant difference.^9^ Diabetic women have insulin resistance with a resultant elevated level of insulin which causes a fall in the concentration of sex hormone-binding globulin which is a carrier of oestrogen.^4^ The high circulating increased free oestrogen causes a resultant ET as observed in this study; however there was no statistical significance between duration of diabetes mellitus and ET among participants corroborating previous report.^4^

This study concludes that parity, number of years passed since menopause and the presence of chronic medical disorders like hypertension or diabetes mellitus were significant determinants of postmenopausal ET in asymptomatic women.

The study recommends that the results of ET in postmenopausal women should be interpreted relative to the woman's parity, BMI and the presence or otherwise of chronic medical disorders. Also, all cases of ET should be followed by endometrial biopsy and histology in asymptomatic women to rule out endometrial pathology.

## Limitations

The study was restricted to one facility and women with both hypertension and diabetes mellitus were not included; this may limit the general application of the results. The general inter-observer variation in the measurement of ET on ultrasonography (though attempts were made to limit this) as well as blind endometrial sampling used for the participant with thickened endometrium compared to hysteroscopy-aided sampling are additional limitations.
